# Interdisciplinary Perspectives on Health Literacy Research Around the World: More Important Than Ever in a Time of COVID-19

**DOI:** 10.3390/ijerph17093010

**Published:** 2020-04-26

**Authors:** Tetine Sentell, Sandra Vamos, Orkan Okan

**Affiliations:** 1 Office of Public Health Studies, University of Hawai’i at Mānoa, Honolulu, HI 96822, USA; 2 School of Interdisciplinary Health Programs, Western Michigan University, Kalamazoo, MI 49008, USA; 3 Interdisciplinary Center for Health Literacy Research, Bielefeld University, 33615 Bielefeld, Germany

**Keywords:** health literacy, infodemic, education, health education, public health, global, trend, COVID-19, interdisciplinary, perspectives, equity

## Abstract

As we write our editorial for a health literacy special issue in the midst of the international COVID-19 crisis, we take this opportunity to highlight the importance of individual, community, and population health literacy. We are not only in a “pandemic” but also an “infodemic”. Health literacy is more important than ever in the face of these global health threats, which have impacted outcomes across the levels of the socio-ecological model (SEM), including individual health behaviors, family relationships, organizational behavior, state policy-making, national mortality statistics, and the international economy in the span of weeks. Our special issue sought to pull together interdisciplinary threads guided by two principles. The first was defining health literacy as essential skills and situational resources needed for people to find, understand, evaluate, communicate, and use information and services in a variety of forms across various settings throughout their life course to promote health and wellbeing. The second was the idea that enhancing health literacy in populations and systems is critical to achieving health equity. In this time of public health need across traditional borders, the inter-sectoral and international perspectives of special issue articles are more urgent than ever. A greater understanding, appreciation, and application of health literacy can support policy action on multiple levels to address major public health challenges. Health literacy should be built deliberately as a population-level resource and community asset. We have summarized the set of articles in this special issue across the levels of the SEM, hoping their thoughtful considerations and interesting findings will help to support global health and wellness and inspire future research, policy, and practice in this global public health emergency and beyond.

## 1. Introduction

Our international perspectives health literacy special issue of the International Journal of Environmental Research and Public Health (IJERPH) sought to attract interdisciplinary papers guided by two principles. The first was defining health literacy as essential skills and situational resources needed for people to find, understand, evaluate, communicate, and use health information and services in a variety of forms across various settings throughout their life course to promote health and wellbeing [1,2]. The second was the idea that enhancing health literacy in populations and systems is critical to achieving health equity. 

As we write this special issue editorial in the midst of the global health threat of COVID-19, individual, community, and population health literacy are more important than ever [3,4,5,6,7]. Personal health behaviors, family relationships, organizational actions, state policy, national mortality statistics, and the international economy have changed in the span of weeks because of decision-making influenced by, and influencing, health literacy. Critical health literacy and digital health literacy are urgently needed by both the citizenry and policy makers to synthesize, analyze, and appraise the vast amount of urgent, complex, and even conflicting information from virologists, epidemiologists, data modelers, doctors, nurses, health departments, and the media [3,5,6,7]. Health literacy capacities allow us to be well-informed about risks, resources, and recommendations and, ideally, to act in solidarity-based behaviors to achieve public health [3,4,7]. It is increasingly clear that social responsibility and solidarity may be key outcomes of health literacy, helping to combat decisions and policies that go against current COVID-19 evidence and best practice public health guidelines [4]. Similarly, and ever more critical, is the concern for health equity. The most profound consequences of the decision made in this health crisis are expected to fall hardest on the most disadvantaged populations [8,9].

We do not live in a vacuum, but rather our individual and collective behaviors and actions are influenced by changing personal and environmental factors. The socioecological model (SEM) [10], describes five levels of influence: individual (e.g., knowledge, attitudes, skills), interpersonal (e.g., families, friends, co-workers, social networks), organizational (e.g., organizations, social institutions, workplaces, schools), the larger community (e.g., the relationship between organizations working together), and public policy (e.g., international, national, state/provincial local laws and regulations). The need for health literacy skills and considerations across all these levels of influence grows increasingly acute. The importance of cross-sector and cross-national health literacy conversations in this time of international crisis are clear, as both the disease and the evidence needed for solutions have rapidly crossed global borders. 

Critical, urgent health information around COVID-19 arrives now every moment from a variety of sources and perspectives [11,12,13,14,15,16,17,18], from personal messages from a health care provider in scrubs and a mask somewhere across the globe that have gone viral on Facebook, to local media, governments, and organizations trying to contextualize the response in a relevant way for their communities. Sorting through the information deluge to find relevant and reliable information to inform the decisions of individuals, families, and communities, and, equally importantly, for health care organizations and governments, is deeply challenging. Adding to complexity to this are divergent opinions, outright hoaxes, and political posturing. We are not only in a “pandemic” but also an “infodemic” [19]. This “global epidemic of misinformation—spreading rapidly through social media platforms and other outlets“ [19] has already had devastating consequences to public health, wellbeing, and economies across the world. Increasing the possibility for confusion in this global pandemic, different countries have implemented different mitigation measures. These differences make finding reliable information even more problematic. Health literacy is even more challenging when knowledge on the topic is incomplete, changing and context dependent.

Yet, at a time like this, health literacy is required around the world to resist the infodemic and to allow individuals to trust and act upon reliable information, recommendations, and advice. This involves people applying a range of skills to make sense of health information and services available even in rapidly changing situations and contexts [2]. Trusted sources must provide reliable and timely information that is context relevant, easy to access, easy to understand, easy to take away, and easy to use. 

Numeracy, the ability to use and understand numbers, is also important. As we are blitzed with news stories involving large, shifting COVID-19 statistics, including actual and projected counts of confirmed infections and deaths every day, this can become overwhelming. The practice of translating statistics and restating data into a meaningful context by experts and advocates for public health action [20,21], also known as “social math”, can support health literacy and numeracy. This approach involves breaking down the data in terms of time or place, making comparisons with familiar things, or personalizing/localizing numbers to make them more digestible and meaningful [20,21]. Infographics and graphs can provide a meaningful way to present data. Social math goes even further to “tell a story” with the data (often including infographics and visuals) to make the point. 

Officials and experts have been advocating action to “flatten the curve”, a message that has reached mainstream consciousness. The “flattening the curve” graphics have provided a simple-to-understand way of visualizing that a slower infection rate means less stress on the health care system. “Physical distancing” has also been a strong and effective message as a protective measure to slow the infection rate when implemented early. These are based on complex research, but also provide clear messages intended for action. One graphic that went viral was from Dr. Singer at the University of California, San Diego, and art director Gary Warshaw, who depicted the complex math in a simple way; specifically, that if one person transmits the infection to an average of 2.5 people, and those 2.5 people each transmit to another 2.5 people and so on, that within 30 days 406 people would be infected [22]. 

While graphs and infographics can present data, in order to build individual and community health literacy and numeracy, we must also continue to tell stories to compliment this data for clear public messaging and to explain risk to those who are not familiar with the language of charts, statistics, and graphs. Visuals alone may not be enough for many to understand how the world works now or what should, and can, be different as part of the solution [23]. People may still wonder and have different interpretations and questions. Figures and statistics need to be part of ongoing conversations that include meaningful framing [24], local context, and personal stories [25] to build health literacy. These may require different methods of communication to visualize the spread [26] or to show the powerful impact of one person’s decision on community risk [27]. 

We are dependent on each other to protect ourselves, loved ones, and communities from COVID-19 and to cope with the illness if it arrives. Services and service providers are also critical. This includes not only primary and emergency health care, but also access to life-sustaining supplies, food, income payments, and housing. Health and social services resources are continuing and, in many cases, growing to respond to this emergency, but must remain dynamic in response to the changing information. Organizations may be stretched too thin delivering actual services to do comprehensive outreach around availability and changes in scope, timing, or eligibility. Yet access to these medical, public health, and social services can be a life-or-death matter to individuals, their neighbors, and communities. Health literacy includes the ability to access and use these services as needed [2]. 

In the 2000s, metaphors like silent epidemic [28,29] and silent killer [30] emerged and were used to figuratively describe the consequences of limited health literacy on individuals and whole economies. The current COVID-19 crisis could sound the alarm, bringing health literacy to the forefront of global conversation and revealing it to be a loud pandemic and explicit killer. Limited health literacy is visibly propelling the spread of COVID-19. For instance, young people defiantly participating in spring break in Florida in March 2020 in spite of widespread calls for physical distancing [31], or Italians speaking to “themselves of 10 days before” about how they wished they had taken calls for precautionary measures more seriously [32]. Higher health literacy, in this case, is coming at an extraordinarily high price for individuals, communities, and governments, who are now calling upon others to heed their lessons [31,33]. 

Practically, the communication surrounding the events of this pandemic in a wide range of political, health, media, and social channels has shown the importance of Nutbeam’s [34,35] communicative and critical health literacy concepts as key tools in slowing down and containing the virus until a treatment and vaccination have been found. The COVID-19 outbreak is health literacy’s test to demonstrate that the concept *is* among the most critical personal and environmental approaches of the 21st century to population health and wellbeing. Only a year ago, Nutbeam [36] wrote a retrospective entitled “Health Education and Health Promotion Revisited”, highlighting the paradigm shift in the way in which public health problems are conceptualized and addressing the move from chronic diseases to new emerging threats such as SARS and Ebola. The concept of health literacy is adaptable to the challenges of our modern times to support the “voluntary changes in behavior” in the “new public health” paradigm while always considering the importance of context.

The need for the effective dissemination of reliable information around COVID-19 facts, precautions, and public health justifications is increasing daily in many communities and across communities as, at the time of writing, people could still travel to return home across states and nations. The need for this information to be given consistently in multiple languages and across a variety of contexts is a major issue for health equity [37] now and in the future. Yet clear language and universal precautions remain critical, as even many people who are well-educated are panicked, confused, or thinking that information does not apply to them [38,39,40]. The ability to even identify reliable sources can be complicated [41]. 

A US-based national coalition of medical and health students, called the COVID-19 Health Literacy Project [42], was recently launched in response to citizen and patient needs for reliable and easy-to-understand information. The project was started by Pooja Chandrashekar and helps vulnerable individuals and communities to protect from and cope with COVID-19 by creating and providing fact sheets and materials in multiple languages. The Lancet [43] has launched their COVID-19 Resource Centre to provide a platform for disseminating and exchanging up-to-date research results to support both health workers and researchers in the fight against this novel disease. Similarly, the New England Journal of Medicine [44] is engaged with research on COVID-19, among efforts by many others.

Not only are scientific organizations providing key resources, but so are major social media entities. Instagram and Facebook are providing useful resources to reduce the misinformation around this critical issue [45], but information and messaging remain complex, inconsistent, and often confusing to many [3,19]. Many have been turning to Google in this time of emergency, and coronavirus became the biggest trend in Google search history due to people looking for information and answers [46]. Consequently, Google launched its separate COVID-19 information site and new search experience for coronavirus queries [47]. This new site is dedicated to education, prevention, and locating resources with the intent to provide people with access to information, safety tips, and search trends related to COVID-19. For example, the top Google trending questions in the US include: (1) How many cases of coronavirus?; (2) Where to get tested for coronavirus near me?; (3) What is coronavirus?; (4) When will coronavirus peak in the US?; (5) How to self-isolate in a shared house? [39]. As the breakout spreads, people want to know how to protect themselves and their families while preparing for uncertainty. Providing reliable, easy-to-understand information in response to these basic, urgent queries is critical. Additionally, individuals’, families’, and communities’ health literacy needs include the awareness and ability to access health information not just about health system functioning and public health messaging but also how to find information about critical matters such as food, child care, workplace safety, and unemployment benefits in the swirl of information that is changing regularly. In the face of a crisis, such as the current COVID-19 pandemic, we are reminded that investing in education for health literacy across the life course is a global resource and community asset. 

As we write this article, COVID-19 has impacted 192 out of 195 countries; this is information that can be seen in one of the critical, innovative health communication/data visualization tools that have emerged in response to this crisis and can support global health literacy: https://ncov2019.live/. Another COVID-19 dashboard has been provided by the Center for Systems Science and Engineering (CSSE) at Johns Hopkins University (JHU) [48], which has evolved into a global data and statistics hub on coronavirus, with many countries having launched similar dashboards to showcase their national and local data. This global health experience, which is also intensely local and personal, will provide important information for future studies. Bielefeld University’s Centre for Interdisciplinary Health Literacy Research is involved in two online surveys on health literacy and COVID-19 information and information behaviour (on adults together with the National Action Plan Health Literacy initiative and Hertie School of Governance; on university students together with Fulda University of Applied Sciences [49]) in Germany. Similar surveys are occurring in Vietnam and Austria. 

## 2. This Special Issue

In this time of crisis, the inter-sectoral, international goals of this special issue are more urgent than ever. Similarly, the resources, findings, and considerations they bring to us are even more necessary to help support global health and wellness and to identify lessons, gaps, and recommendations. Through our call for abstracts, our specific topic goals were the following:

- Research considering cross-country comparisons and/or cross-sector collaborations;
- Research promoting or evaluating a system’s approach to health literacy;
- Research that captures the contemporary discussion on theory, models, definitions, and concept analyses for different populations and age groups, including insights from other scientific disciplines;
- Research and practice initiatives on the social function of health literacy, social contexts, social health literacy, and distributed health literacy;
- Research and practice results that move global, national, or regional policies to action, or the evaluation of those policies in terms of health promotion and the social determinants of health;
- Research considering capacity building and empowerment for health literacy across professions, settings, and systems (such as education, health care, workplace, business, and government).

We received a large number of potential submissions, reflecting the growing interest in the field. Specifically, the final set of articles completing peer-review for this special issue touch upon a number of areas that have resonance in this time and the future, aligning with many levels of the socioecological model. We thus present them here across the levels of the socioecological model: individual (prevention, chronic care); interpersonal (social networks); organizational (health systems, workplaces, schools), larger community (tailoring for culture and context); and public policy. 

Reflecting a growing trend to consider health literacy at levels beyond the individual [50,51], we note how many of our articles are related to issues beyond individual skill or the patient-provider relationship (which, of course, remain critical) to focus on organizational health literacy capacity and solutions or health literacy as experienced in a social context.

## 3. Individual Level: Prevention and Chronic Care

Reflecting the worldwide epidemiological trends of the growing disease burden, care for chronic disease was a critical focus, especially cardiovascular disease [52]. Cabellos-García et al. [53] give us new insights in “Relationship between Determinants of Health, Equity, and Dimensions of Health Literacy in Patients with Cardiovascular Disease” in the development of suitable strategies to reduce inequity. In “Health Literacy among People in Cardiac Rehabilitation: Associations with Participation and Health-Related Quality of Life in the Heart Skills Study in Denmark” by Aaby et al. [54], the authors help us to understand the associations between health literacy and cardiac rehabilitation outcomes. Muscat et al. [55] give information on those engaged in a chronic disease prevention program in “The Impact of the Chronic Disease Self-Management Program on Health Literacy: A Pre-Post Study Using a Multi-Dimensional Health Literacy Instrument”. 

A number of articles also focused on prevention. In “Health Literacy as Communicative Action—A Qualitative Study among Persons at Risk in the Context of Predictive and Preventive Medicine”, Harzheim et al. [56] consider the role of predictive and preventive medicine in health literacy. Rudolf et al. [57] provide objective information that health literacy has a relationship with physical activity in “Influence of Health Literacy on the Physical Activity of Working Adults: A Cross-Sectional Analysis of the TRISEARCH Trial”.

## 4. Interpersonal Level: Social Networks

Health information is spread, digested, and created in communities and social networks. Several articles help provide insight on these perspectives. Kendir and Breton [58] do so in “Health Literacy: From a Property of Individuals to One of Communities” as they consider this in more detail. Lorini et al. [59] consider a specific topic on the influence of health literacy as a distributed resource available within an individual’s social network in “Health Literacy as a Shared Capacity: Does the Health Literacy of a Country Influence the Health Disparities among Immigrants?” The article by Amoah [60], “The Relationship between Functional Health Literacy, Self-Rated Health, and Social Support between Younger and Older Adults in Ghana”, considers which aspect of social support (instrumental, informational, and emotional support) is responsible for health literacy by comparing two groups in an understudied context. In “Considering Health Literacy, Health Decision-Making, and Health Communication in the Social Networks of Vulnerable New Mothers in Hawai‘i”, Sentell and colleagues [61] consider the importance of health literacy in the decision-making networks of new mothers, and how to measure this shared capacity using social network analyses. Bessems et al. [62] created and tested small-group nutrition education intervention for adults with low socioeconomic status and small incomes in “The Effectiveness of the Good Affordable Food Intervention for Adults with Low Socioeconomic Status and Small Incomes”. 

## 5. Organizational Level: Health Systems

The growing research topic of organizational health literacy was the level of the social ecological model with the most articles. These articles include “Improving Organizational Health Literacy Responsiveness in Cardiac Rehabilitation Using A Co-Design Methodology: Results from The Heart Skills Study” by Aaby et al. [63], which considers how to improve organizational health literacy responsiveness in cardiovascular care. Trezona et al. [64] provide a relevant self-assessment tool for organizational health literacy in “Field-Testing and Refinement of the Organisational Health Literacy Responsiveness Self-Assessment (Org-HLR) Tool and Process”. Kaper et al. [65] consider the sustainable changes and longitudinal outcomes of organizational health literacy efforts in “Implementation and Long-Term Outcomes of Organisational Health Literacy Interventions in Ireland and The Netherlands: A Longitudinal Mixed-Methods Study”. As in many studies, this considers two different health contexts and the need to address health inequality. In “A Scoping Review on How to Make Hospitals Health Literate Healthcare Organizations”, Zanobini et al. [66] consider the concept of health literacy as a trait of organizations providing health and social services. 

## 6. Organizational Level: Schools, Students, and Adolescents

A number of our studies focused on the young, especially considering the health literacy capacity of the school setting, the organization in which youth spend much of their time. Health literacy and many of its determinants are well studied in adults but under researched in children and adolescents. In “The Online Survey for the Assessment of Generic Health Literacy among Adolescents in Germany (GeKoJu): Study Protocol” by Loer et al. [67], the authors describe a study to provide a sound data basis on adolescent health literacy in Germany to support health literacy goals. Fretian et al. [68] consider the measurement of health literacy in children in “Exploring Associated Factors of Subjective Health Literacy in School-Aged Children in Germany”. Sukys et al. [69] similarly consider this issue in the context of an understudied population in “Subjective Health Literacy among School-Aged Children: First Evidence from Lithuania”. Parents are shown to be key to health literacy in young people in “How Did Parents View the Impact of the Curriculum-Based HealthLit4Kids Program Beyond the Classroom?” Nash et al. [70] consider parents’ perspectives on the HealthLit4Kids program, which aims to build health literacy in a participatory and contextually relevant way. Klinker et al. [71] find that “Health Literacy is Associated with Health Behaviors in Students from Vocational Education and Training Schools: A Danish Population-Based Survey”, supporting an important and changeable intermediary determinant of health equity in a population where many students come from a low socio-economic status. Dadaczynski et al.’s [72] study “The Role of School Leaders’ Health Literacy for the Implementation of Health Promoting Schools” considers the promotion of health literacy as an urgent goal in public health and education and describes a need for it to be integrated in the school context as a component of the holistic health promoting school (HPS) approach. In “Potentials of School Nursing for Strengthening the Health Literacy of Children, Parents and Teachers”, Buhr et al. [73] conducted a pilot study in Germany, finding promisingly that school nurses may contribute to strengthening health literacy within the school setting. Rathmann and colleagues [74] consider the organizational health literacy in facilities for people with disabilities in their pilot mixed-methods study: “Organisational Health Literacy in Facilities for People with Disabilities: First Results of an Explorative Qualitative and Quantitative Study”. Domanska et al. [75] provide new evidence around self-reported health literacy in adolescence in “Development and Psychometric Properties of a Questionnaire Assessing Self-Reported Generic Health Literacy in Adolescence”.

## 7. Organizational Level: Workforce in Health Care and Beyond

Considerations of the workforce remain vital to understanding and addressing health literacy needs. Rowlands and colleagues [76] provide useful guidance in sharing “Evidence-Based Development of an Intervention to Improve Clinical Health Literacy Practice”. Juvinyà-Canal and colleagues [77] give us perspectives in “Health Literacy Among Health and Social Care University Students” to understand better the caregiver pipeline, which can support individual and organizational health literacy and direct interventions. In “Effectiveness of a Comprehensive Health Literacy Consultation Skills Training for Undergraduate Medical Students: A Randomized Controlled Trial”, Kaper et al. [78] provide future doctors with a larger scope of capacities to strengthen a patient’s autonomy, participation, and self-management abilities. Koduah et al. [79] consider how sociocultural factors affect the health literacy practices of nurses, especially in low-income countries, in “’I Sometimes Ask Patients to Consider Spiritual Care’: Health Literacy and Culture in Mental Health Nursing Practice”. Considering a community not often included in health literacy, Coman et al. [80] focus on agricultural workers in “Educational Interventions to Improve Safety and Health Literacy Among Agricultural Workers: A Systematic Review”. This can improve farmers’ health and quality of life. In their systematic review, “The Agreement between Patients’ and Healthcare Professionals’ Assessment of Patients’ Health Literacy—A Systematic Review”, Voigt-Barbarowicz and Levke Brütt [81] investigate the agreement between the patients’ and health care providers’ assessments of patients’ health literacy. In “Using the Health Literacy Questionnaire (HLQ) with Providers in the Early Intervention Setting: A Qualitative Validity Testing Study”, Leslie et al. [82] consider the health literacy of interdisciplinary early intervention providers who are optimally placed to build the health literacy capacity of caregivers and whose health literacy has not previously been measured.

## 8. Community Level: Tailoring by Culture/Topic/Population Needs

Several articles consider the critical importance of tailoring by community, culture, and need, including several that provide an international comparison. In “Adolescent Health Literacy in Beijing and Melbourne: A Cross-Cultural Comparison”, Guo and colleagues [83] consider adolescent health literacy from a cross-cultural perspective. In “Factors that Influence the Former Soviet Union Immigrants”, Kostareva et al. [84] consider the unique health literacy needs of those from a specific cultural context in whatever country they arrive. In “Inclusion of People with Intellectual Disabilities in Health Literacy”, Latteck and Bruland [85] present findings from three different studies on the health literacy of particular vulnerable groups. Ehmann et al. [86] give information to guide future work in “The Relationship between Health Literacy, Quality of Life, and Subjective Health: Results of a Cross-Sectional Study in a Rural Region in Germany”, which will be of particular relevance to a regional integrated healthcare system. Sykes et al. [87] give insights on health literacy on the topic of infertility in “Multidimensional eHealth Literacy for Infertility”, considering the concept of eHealth literacy in individuals and couples in relation to infertility. In “Validation of the Short-Form Health Literacy Questionnaire (HLS-SF12) and Its Determinants among People Living in Rural Areas in Vietnam”, Duong et al. [88] validate the use of a comprehensive short-form health literacy (HL) survey tool (HLS-SF12) in Vietnam. The study by Chakraverty et al. [89], “Gender-Specific Aspects of Health Literacy: Perceptions of Interactions with Migrants among Health Care Providers in Germany”, explores the views of health care professionals on how gender affected their interactions with migrant patients. In “Dementia Literacy in the Greater Bay Area, China: Identifying the At-Risk Population and the Preferred Types of Mass Media for Receiving Dementia Information”, Leung and colleagues [90] consider the mass media preferences for receiving dementia information of community-dwelling adults in four cities (Hong Kong, Guangzhou, Macau, and Zhuhai) of the Greater Bay Area of China. 

## 9. Policy Level: Comparing Options Across Countries and the Opportunity for Impactful Change

Hofer-Fischanger et al. [91] give us new insights into active living and health literacy in “Health Literacy and Active Transport in Austria: Results from a Rural Setting”, providing useful guidance. In “Making a Case for ‘Education for Health Literacy’: An International Perspective”, Vamos et al. [92] describe critical perspectives on merging health literacy in the educational sector across contexts and how to bring policy into practice. Mansfield et al. [93] should provide valuable policy-level perspectives in “Integrating a Health Literacy Lens into Nutrition Policy in Canada”. 

## 10. International Research Evidence

Perhaps most importantly, this special issue brings together researchers across the globe as well as across disciplines. We have research from low, middle- and high-income countries. We have research from Europe (e.g., Lithuania, Germany, Austria, Denmark, Sweden), North America (e.g., USA, Canada), and Asia (e.g., Vietnam, Taiwan) along with research from Africa (Ghana), which is understudied in health literacy. We also consider cross country comparisons—comparing Beijing and Melbourne in the study by Guo and colleagues [83] and the health literacy of former Soviet Union immigrants across the globe in the study by Kostareva et al. [84]. 

The global community of researchers and scientists is working together to build actionable, meaningful evidence to support shared goals of health and community well-being. Even as we close borders, we understand that responses and health issues are interconnected across the globe. We model and learn from decisions in other locations, including about health information and what works. We hope this set of articles and their wide-ranging lessons and perspectives inspire other research. Some areas remain underrepresented in health literacy, including the Pacific and many parts of Africa. Some authors of initial article proposals were unable to complete their papers in these times of regional crisis (Lebanon) and global stress. In the future, we look forward to seeing more innovative work concerning the topic of interest to this special issue: cross-country comparisons and cross-sector collaborations; systems’ approaches to health literacy; new theories, models, definitions, and concept analyses; innovative research on health literacy in social contexts and health literacy policies in action; health literacy research considering the capacity-building and empowerment of health literacy across professions, settings, and systems. These efforts will all help build health literacy locally, nationally and globally. 

Finally, as editors from three countries (U.S., Canada, and Germany) who have worked closely together for over a year to produce this special issue using virtual tools that would not have been possible even ten years ago, we can attest to the ability to build knowledge, connections, and productivity in new information environments. Many of our most treasured colleagues live across the world. This time may open both new realities but also new pathways for health communication to build and support health literacy towards better individual and community health. 
